# Poisoning of Cattle with Nitrate of Sodium

**Published:** 1895-02

**Authors:** Jas. B. Paige


### POISONING OF CATTLE WITH NITRATE OF SODIUM.

BY JAS. B. PAIGE, D.V.S.

From the fact that large quantities of strong chemicals are being used as fertilizers by our farmers, we believe the case as detailed below is of sufficient interest to warrant its publication, showing what the results may be if they are not carefully handled.

On May 31st of last year in company with Dr. J. B. Lindsey, vice-director of the Massachusetts Agricultural Experiment Station, I had occasion to investigate a case in which eleven cows of a herd of fifteen had died as a result of giving nitrate of sodium in place of chloride of sodium.

The history of the case given by the owner was as follows: The fifteen cows had been in the pasture during the day. Between five and six o'clock in the afternoon they were driven to the stable, and all at that time appeared perfectly well.

When in the stable a man, a native of Poland, whose work it was to care for the cattle, gave to all except two what he supposed to be common coarse-fine salt, thoroughly mixed with a small quantity of wheat bran. Two had no bran, but in place had clear salt. So far as is known all of the animals ate heartily of the salt.

About eight o'clock of the same evening the noise made by the cows attracted the attention of one of the men about the place, who upon investigating the cause of the disturbance found that they appeared to be uneasy and showed symptoms of suffering pain. Within an hour or two several had died.

A practitioner of human medicine was called to treat the animals, and, thinking the trouble due to arsenic, prescribed dialized iron, raw eggs, etc. The treatment seemed to avail nothing, and before morning eleven of the herd were dead. Of the four which lived, three showed all of the symptoms exhibited by those which had died; the fourth did not appear to be affected at all, and the question arises as to whether she was given any of the salt, as none was found in or about her manger the following day.

The symptoms exhibited by the affected animals, according to the statement of the physician, were evidence of severe abdominal pain, throwing the head from side to side, continual stepping, whisking of the tail, later twitching of the muscles, followed by paralysis of the posterior parts of the body, falling, inability to rise when down, resting of the head upon the sides and occasionally pressure of the nose upon the ground. In nearly all of the cases the anus and vulva became swollen and dark in color. In some, purgation existed with frequent straining; thin feces, not colored with blood, were passed. In none was diuresis a marked symptom. Shortly before death the respiration was hurried and labored, especially so in some. In general the symptoms indicated intense abdominal pain, dyspnœa, and paralysis, particularly of the posterior extremities.

On the day following the eating of the salt, the three living cows that had during the night shown the same symptoms as those which had died did not appear to be suffering to any great extent. An examination showed the temperature to be only slightly elevated, the pulse nearly normal, the mucous membrane but little injected. Food was refused by all. While the feces passed were thin and watery, they were not abnormally abundant, neither were the functions of the kidneys disturbed to any extent. Slight muscular weakness or paralysis existed in the posterior extremities. So far as known the recovery of these animals was complete.

Autopsies made the following afternoon (eighteen hours after death) upon two of the cows which had died first and had not been given drugs revealed the following condition: Parts of the mucous membrane of the rectum and of the vagina were everted and swollen and of a bluish-black color. Upon opening the abdominal cavity numerous ecchymotic spots were found on the peritoneum and viscera. The mucous membrane of the first and second stomachs near the œsophagus was thickened and red in color, and in these parts it was easily detached from the tissues beneath.

The lungs were semi-solid, dark in color, and the bloodvessels filled with dark blood. Scrapings from a cut section showed the presence of air in the air cells. The left side of the heart contained but little blood, while in the cavities of the right large, black semi-fluid clots were found. All other organs of the body were normal.

The condition of the heart and lungs found upon post-mortem examination would indicate that death resulted from asphyxia.

A chemical examination by Dr. Lindsey of samples of material collected from the mangers, from a pail partly filled with a mixture of bran and salt that had been taken from the mangers, from the ground near the pig-pen and barn door, from a box in a shed near by, and from a barrel in the same shed in which the nitrate of sodium was kept, showed in every sample the presence of large quantities of nitrate of soda but no chloride of sodium.

A chemical examination of a part of the contents of the stomach failed to reveal a trace of arsenic, lead, or other mineral poison.

In the *Veterinarian* for 1876 there are several articles translated from the *Archives Vétérinaires* by Professor Tuson reporting cases similar to this.

One was that of a horse that had drunk ten and one-half pints of a solution of nitrate of soda containing twenty-five and three-fourths ounces of the salt. This animal was seen six hours after taking the soda in solution, and it presented many of the symptoms noticed in these cattle. The administration of strong infusions of coffee and alcohol and irritant clysters relieved the animal, and in a few days he completely recovered from the effects of the poisonous salt.

The second case was that of a five-year-old gelding that had drunk from a pool in which nitrate of soda bags had been washed; he showed the same symptoms and responded to the same treatment as the animal just mentioned.

In the third case a four-year-old mare drank ten and one-half pints of a solution of nitrate of soda, containing between seventeen and one-half and twenty-one ounces of the salt. In a few hours she was attacked with violent colic, which was relieved by the use of clysters of coffee and alcohol. She made a good recovery.

In the second article published there are recorded cases of poisoning among cattle from nitrate of soda. Four bags of nitrate of soda had been placed at equal distances on a grass field, and had been allowed to remain there about five hours before being applied. During this time some of the salt had become washed from the bags upon the ground by a slight fall of rain. Three days afterward cows were turned into the pasture and obtained the salt by licking it from the ground where the bags had been. They afterward showed symptoms of poisoning.

The last case reported in these articles is as follows: "Two hundred bags which had contained nitrate of soda were washed in four large tubs full of water, close to which the cattle passed on the way to the meadows. At about nine A.M. forty-six cows and heifers drank of this solution of the nitrate . . . . At three P.M. the forty-six cows and heifers returned to the farm. At four P.M. a heifer died suddenly, and at five P.M. another heifer succumbed."

Although the death of only two heifers occurred, many of the other cattle that drank of the soda solution showed marked symptoms of its effects. The death of these two is accounted for by the writer from the fact that they were the first ones to leave the stable, and consequently had more time to drink at the tubs than did any of the other animals which followed.

